# Characterization of CD8 T Cell-Mediated Mutations in the Immunodominant Epitope GP33-41 of Lymphocytic Choriomeningitis Virus

**DOI:** 10.3389/fimmu.2021.638485

**Published:** 2021-06-14

**Authors:** Mark Smyth, Kseniya Khamina, Alexandra Popa, Venugopal Gudipati, Benedikt Agerer, Alexander Lercher, Lindsay Kosack, Lukas Endler, Hatoon Baazim, Csilla Viczenczova, Johannes B. Huppa, Andreas Bergthaler

**Affiliations:** ^1^ CeMM Research Center for Molecular Medicine of the Austrian Academy of Sciences, Vienna, Austria; ^2^ Center for Pathophysiology, Infectiology and Immunology, Institute for Hygiene and Applied Immunology, Medical University of Vienna, Vienna, Austria

**Keywords:** viral infection, lymphocytic choriomeningitis (LCMV), CD8 T cell, immune evasion, mouse model, CTL escape mutation

## Abstract

Cytotoxic T lymphocytes (CTLs) represent key immune effectors of the host response against chronic viruses, due to their cytotoxic response to virus-infected cells. In response to this selection pressure, viruses may accumulate escape mutations that evade CTL-mediated control. To study the emergence of CTL escape mutations, we employed the murine chronic infection model of lymphocytic choriomeningitis virus (LCMV). We developed an amplicon-based next-generation sequencing pipeline to detect low frequency mutations in the viral genome and identified non-synonymous mutations in the immunodominant LCMV CTL epitope, GP33-41, in infected wildtype mice. Infected Rag2-deficient mice lacking CTLs did not contain such viral mutations. By using transgenic mice with T cell receptors specific to GP33-41, we characterized the emergence of viral mutations in this epitope under varying selection pressure. We investigated the two most abundant viral mutations by employing reverse genetically engineered viral mutants encoding the respective mutations. These experiments provided evidence that these mutations prevent activation and expansion of epitope-specific CD8 T cells. Our findings on the mutational dynamics of CTL escape mutations in a widely-studied viral infection model contributes to our understanding of how chronic viruses interact with their host and evade the immune response. This may guide the development of future treatments and vaccines against chronic infections.

## Introduction

The action of cytotoxic T lymphocytes (CTLs) is a key means by which the immune system controls viral infections (1, 2). CTLs recognize peptides presented by major histocompatibility complex I (MHCI) (3). During viral infection, MHCI complexes present virus-derived peptides on the cell surface (3, 4), which are detected by CTLs *via* the T cell receptor (TCR). Viral epitopes are restricted to one of the three types of MHCI, each type containing a unique α chain. In mice, the α chain is encoded by the H-2D, H-2K and H-2L loci (5). Binding of the TCR to the viral peptide-MHCI complex, together with sufficient costimulatory signals from receptor/ligand interactions and cytokines, results in proliferation and migration of the CTLs to the site of infection (2). CTLs initiate targeted cell death, by apoptosis, of infected cells through the secretion of perforin and granzymes and/or binding of Fas ligand to Fas receptors on the infected cell (6, 7). Due to their cytotoxic nature, CTL activity constitutes a direct antiviral selection pressure and is considered essential to control viral infections (8).

Increased mutation rates of CTL epitopes are a classical mechanism by which chronic viruses like lymphocytic choriomeningitis virus (LCMV), hepatitis C virus (HCV) and human immunodeficiency virus (HIV) may evade CTL-mediated clearance (9–12). These escape mutations commonly occur in or adjacent to the epitopes and normally affect one of the three following processes: the processing of viral peptides, the display of viral peptide on the MHCI molecule and the recognition of the MHCI-peptide complex by the TCR (13, 14). Anchor residues facilitate the binding of peptides to the MHCI molecule, where they occupy binding pockets (5). Accordingly, if an anchor residue is mutated, this often leads to the reduction or the ablation of the binding of the peptide to the MHCI molecule (15).

Experimental infections with LCMV are a benchmark model for studying chronic viral infection and CTL responses (16). The virus-encoded CTL epitopes are well-characterized for the infection of H-2D^b^/H-2K^b^ restricted C57BL/6J wildtype (WT) mice (17). This model enabled the discovery of the first viral CTL escape mutation (18) and it has provided additional valuable insights into viral immune evasion by non-synonymous mutations in CTL epitopes (19–22).

Although several CTL escape mutations have been identified in LCMV, the emergence dynamics of these mutations have not been comprehensively studied. Here, we infected C57BL/6J WT mice with the chronic LCMV strain, Clone 13, and analyzed viral sequences from the spleens of these mice using a next-generation sequencing approach. We focused on the immunodominant viral epitope GP33-41 (23) and employed complementary experimental approaches to modulate the CTL selection pressure and elucidate the dynamics of emerging viral mutations. Selected mutations were characterized by determining peptide binding to recombinant H2-D^b^ molecules as well as by characterizing mice infected with reverse genetically engineered viruses carrying identified mutations in the GP33-41 epitope.

## Materials and Methods

### Mice

All animal experiments were conducted in compliance with the respective mouse licenses with the protocol numbers BMWFW-66.009/0318-II/3b/2012, BMWFW-66.009/0199- WF/V/3b/2015 and BMWFW-66.009/0361-WF/V/3b/2017 in accordance with the Austrian law for animal experiments (TVG, BGBl. Nr. 501/1989 i.d.F. BGBI. I Nr. 162/2005). All animal licenses were approved by the relevant institutional ethics boards in accordance with institutional guidelines.

All mice used in these experiments were on a C57BL/6J background, age-matched and male. Genetically modified mice included P14 TCR-transgenic (24) and *Rag2^-/-^* (25) mice. The mice were housed under specific pathogen free conditions at the Institute of Molecular Biotechnology, Vienna, Austria, the Anna Spiegel animal facility of the Medical University of Vienna, Vienna, Austria and the animal facility of the Department of Biomedical Research of the Medical University of Vienna, Vienna, Austria.

### Viruses

All mice were infected intravenously (i.v.) *via* the tail vein with 2 x 10^6^ focus forming units (FFUs) of the Clone 13 strain of LCMV or the respective viruses containing mutations in the GP33-41 epitope. All viruses were generated using a reverse genetic rescue system as described previously (26). Virus stocks of passage two were used for infection experiments.

### Tissue Collection

For longitudinal measurements of viral titer blood was collected from the tail vein in 1ml of MEM-Heparin (10U/ml). The MEM was purchased from Sigma-Aldrich (M4655) and the heparin from Ratiopharm. Organs were collected upon whole body perfusion of anesthetized animals with 20ml PBS. For the sequencing experiments spleens were harvested, rinsed in PBS and were frozen in liquid nitrogen for further processing. For measuring viral titers from solid organ samples, organs were harvested in the same way but were collected in 1ml MEM supplemented with 5% fetal calf serum (Invitrogen) and 1% Penicillin-Streptomycin-Glutamine (Thermo Fisher Scientific, 10378016) before being frozen in liquid nitrogen. For flow cytometry analyzes blood and spleen samples were collected and prepared as previously described (27).

### RNA Extraction and cDNA Synthesis

Spleen samples were homogenized in QIAzol lysis reagent (QIAGEN, 79306) using a TissueLyser II (QIAGEN, 85300) and RNA was extracted in accordance with the manufacturer’s instructions. We used the First Strand cDNA synthesis Kit (Thermo Fisher Scientific, K1622) using 1µg of RNA as input and the manufacturer’s standard protocol to synthesize cDNA.

### Amplicon-Based Next-Generation Sequencing Experiments

To amplify a 540bp fragment of the LCMV S segment containing the GP33-41 epitope, the following primers were used: Forward 5’-CGCTTTCCTCTAGATCAACTGG-3’, Reverse 5’- CATTGTTGAAGTCGCAGGATAC-3’. The PCR with Phusion high fidelity polymerase (Thermo Fisher Scientific, F530S) was run for 45 cycles and with a melting temperature of 68°C, in accordance with the manufacturer’s 20µl reaction protocol. Once the PCR was completed the samples were cleaned with AMPure XP beads (Beckman Coulter, A63881) to remove the PCR primers and enzyme. Sequencing libraries were created from these samples using a Nextera XT DNA library preparation kit (Illumina, FC-131-1096) using the manufacturer’s standard protocol. The libraries were sequenced on a MiSeq v2 micro with a 150bp paired end protocol. Base calling was achieved using the Illumina Realtime Analysis software. Data were converted into BAM format using Illumina2bam and demultiplexed using BamIndexDecoder (https://github.com/wtsi-npg/illumina2bam). We obtained an average coverage of 1.5 x 10^5^ reads per sample at the GP33-41 epitope. Read filtering for adapter and primer sequences as well as for quality and length was performed with BBDUK from the BBtools suite (http://jgi.doe.gov/data-and-tools/bbtools). Mapping of paired reads on the combined mm10 (GRCm38/mm10) mouse genome and LCMV genome segments (GenBank: DQ361065.2 and DQ361066.1) was done with the BWA-MEM software package (28) (v 0.7.17) with a minimal seed length of 17. For calling virus variants we realigned the BAM alignment file using the Viterbi method implemented by LoFreq (29) (v 2.1.2). InDel qualities for low frequency variants were called using LoFreq. A reference genome of the short segment of LCMV Clone 13 (GenBank: DQ361065.2) was employed for the variant calling. Variant filtering was performed with LoFreq and Bcftools (30) (v 1.9) using a minimum coverage of 75 reads, a minimum base quality of 10, a minimum mapping quality of 20 and a significance threshold of 0.05 (-q 10 -Q 20 -m 20 -C 75 -a 0.05). The programs SnpEff (31) (v 4.3) and SnpSift (32) (v 4.3) were used for variant annotation.

### Plasmid Titration to Validate Amplicon-Based Next-Generation Sequencing Pipeline

A solution of plasmid containing the whole LCMV S (short) segment with a G->A nucleotide exchange at position 178 of the S segment (the GP34A->T mutation) was serially diluted in a solution of a plasmid containing WT S segment. This was done to produce final solutions containing the percentage of mutant plasmid indicated in **Supplementary Figure 1**. The concentration of total plasmid in these solutions was 10ng/ml. These solutions were then processed using the full next-generation sequencing pipeline described above.

### Predictions of Peptide Binding to H2-D^b^

We used the bioinformatic tool netMHCpan (v 4.0) (33) to predict the binding of peptides to H2-D^b.^ (http://www.cbs.dtu.dk/services/NetMHCpan-4.0) with default settings defining a rank score of <0.5% as strong binding, a rank score of 0.5%-2% as weak binding and a rank score of >2% as little to no binding.

### Splenocyte Transfer Experiment

Whole spleens from P14 TCR-transgenic mice were homogenized by passing them through 40µm filters. The splenocytes were counted and the homogenates were diluted to 5 x 10^7^ splenocytes/ml in PBS. 200µl of this splenocyte dilution was injected intravenously *via* the tail vein so that each mouse received 1 x 10^7^ splenocytes. One day later the recipient mice, as well as WT mice that did not receive splenocytes, were infected with LCMV Clone 13 WT as described above. The mice were sacrificed 40 days after LCMV infection. The details of sacrifice and organ collection are described above.

### Sanger Sequencing

Using the same PCR conditions as detailed above, a 540bp fragment containing the GP33-41 epitope was generated and run on a 1% agarose electrophoresis gel containing 0.0025% ethidium bromide (Sigma, E1510), along with a 100bp ladder (New England Biolabs, N0551S). Excised bands were purified with a gel extraction kit (Qiagen, 28706) using the manufacturer’s protocol and eluting the final products in 40µl DNase/RNase free water. The products were then submitted to Microsynth AG for Sanger sequencing. The percentage of the GP34A->T and GP35V->A mutations in each sample was estimated with QSVanalyzer using -10bp to -5bp as reference bases (34).

### Virus Propagation on BHK-21 Cells

BHK-21 cells were maintained in Dulbecco׳s Modified Eagle Medium (Gibco, 41985-039) supplemented with 10% fetal calf serum (Invitrogen), 2% tryptose phosphate broth (Sigma, T8159), 1% 1M HEPES solution (Sigma, H0887), 1% 100mM sodium pyruvate solution (Sigma, S8636) and 1% Penicillin-Streptomycin-Glutamine (Thermo Fisher Scientific, 10378016) throughout all experiments. 3 x 10^5^ BHK-21 cells per well were plated in 6-well plates and were left overnight at 37°C. The next day the sub confluent cells were infected at a multiplicity of infection (MOI) of 0.01 (assuming 6 x 10^5^ cells per well). For these infections LCMV Clone 13 WT, LCMV Clone 13 containing the GP34A->T mutation or LCMV Clone 13 containing the GP35V->A mutation was used. Three replicate wells were used for each condition. After infection 2ml of media was added to each well. After 6, 12, 24, 48 and 72 hours post infection 1ml of media was taken for further analysis and then 1ml of fresh media was added. At 96 hours post infection, all the media was taken.

### Flow Cytometry Analyzes

All surface and intracellular antibodies used in the flow cytometry analyzes were purchased from Biolegend. All dilutions containing staining reagents (antibodies, tetramers and the viability dye) where made using FACS buffer (2% FCS/PBS). Data were analyzed using FlowJo software (v 10).

GP33-41 tetramers with a PE fluorophore, GP276-286 tetramers with a FITC fluorophore and NP396-404 tetramers with an APC fluorophore were obtained from the NIH Tetramer Core Facility. The samples were first stained with the tetramers (GP33-41 and NP396-404, 1:500; GP276-286, 1:200) at 37°C for 15 minutes. After tetramer staining the samples were stained with anti-CD16/CD32 block antibody (1:200, clone 93) at room temperature for 10 minutes and then 1:200 with surface antibodies: CD3e: Pe-Cy7, clone 145-2C11; CD8a: AF700, Clone 53-6.7 at 4°C for 20 minutes. They were concurrently stained 1:2000 with Fixable Viability Dye; eFluor 780 (ebioscience, 65-0865-18). The samples were washed in FACS buffer and then fixed in 4% formaldehyde solution (Sigma-Aldrich, 252549-1L) in FACS buffer at room temperature for 10 minutes. The samples were then resuspended in 200µl of FACS buffer and analyzed by flow cytometry on a LSRFortessa flow cytometer (Biosciences).

For the splenocyte transfer experiment, the percentage of cells from P14 TCR-transgenic mice in the CD8+ population was calculated as the percentage of the CD45.1+ cells in the CD8+ population. The samples were stained 1:200 with the same surface antibodies as for the tetramer experiments (see above) in addition to a CD45.1 antibody (clone A20, Pacific Blue). For the flow cytometry analysis of P14 TCR-transgenic mice the samples were stained with tetramers and treated with anti-CD16/CD32 block antibody as described above. They were then stained 1:200 with the following surface antibodies: CD8.2b: Pacific Blue, clone 53-5.8; CD44: Brilliant Violet 605, clone IM7 and they were concurrently stained 1:2000 with Fixable Viability Dye: eFluor 780, (ebioscience, 65-0865-18). Samples were then processed and analyzed as described above.

Intracellular cytokine staining was performed as previously described (20). The GP33-41 peptides (KAVYNFATC, KTVYNFATC and KAAYNFATC) were purchased from BonOpus biosciences at a purity of >90%.

### Measurements of Viral Titer

For all relevant samples, (blood, organ pieces, viral stock, media from the BHK-21s cells) viral titer was quantified with focus forming assay as described previously (35).

### Differential Scanning Fluorimetry

The recombinant peptide-MHCI complexes were produced as described previously (36–38). The UV-cleavable peptides for H2-D^b^ and H2-K^b^, ASNENJETM (36) and FAPGNYJAL (39) respectively, were purchased crude (70% purity) from Intavis Peptide Services and were used in all differential scanning fluorimetry experiments. The peptide-MHCI complexes for the differential scanning fluorimetry experiments were made (37) and the experiments performed as described previously (40). The GP33-41 peptides used in **Figures 3D, E** were the same as for the intracellular cytokine experiment (see above). The GP33-41 peptides used in **Supplementary Figure 8** were purchased crude (70% purity) from JPT peptide services. The GP34-43 peptides used in **Supplementary Figure 10** were purchased from BonOpus biosciences at a purity of >90%.

## Results

### Mutations Emerge in the Immunodominant GP33-41 Epitope in LCMV-Infected WT Mice

**Figure 1:**
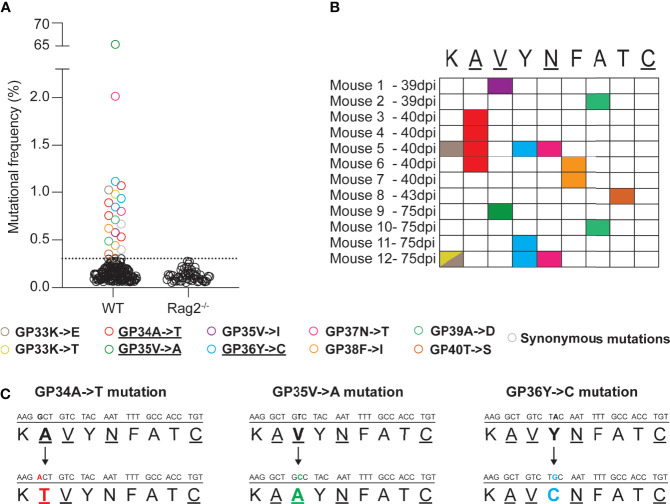
Mutations emerge in the immunodominant GP33-41 epitope in LCMV CL13 WT in infected WT mice. **(A)** Mutations in the GP33-41 epitope identified in WT and *Rag2^-/-^* mice using next-generation sequencing. The data from WT mice represents three, eleven, two and five mice harvested at 39, 40, 43 and 75 days post infection respectively. These data are from six separate experiments. The data for the *Rag2^-/-^* mice represent six and two mice harvested at 40 and 75 days post infection respectively. These data are from two separate experiments. The dotted line indicates the mean +3 standard deviations of the combination of the frequencies of all the mutations in the *Rag2^-/-^* mice at both time points. **(B)** Schematic indicating the type and position, in the GP33-41 epitope, of the mutations identified in mice chronically infected with LCMV CL13 WT. **(C)** Diagram of the mutations identified repeatedly (GP34A->T and GP36Y->C) or at high frequency (GP35V->A) in the WT mice. The underlined amino acids are anchor residues.

Firstly, we wanted to investigate the emergence of non-synonymous mutations in the immunodominant, H2-D^b^ restricted GP33-41 epitope of LCMV (nucleotide positions 175-201 of the S segment) during the course of a chronic infection in WT mice. To recover low frequency mutations, we employed a LCMV-specific amplicon-based next-generation sequencing protocol that allows the identification of low frequency mutations and that we validated by a plasmid titration experiment (**Supplementary Figure 1**). We collected spleen tissue from WT mice that were infected with 2 x 10^6^ focus forming units (FFU) of LCMV Clone 13 at various timepoints (39, 40, 43 and 75 days post infection) and subjected them to our sequencing protocol. We identified at least one non-synonymous mutation in 12 out of 21 infected WT mice, from six independent experiments, in the GP33-41 epitope (**Figures 1A, B**). Ten distinct, possible escape mutations were identified and 60% of which were seen in multiple mice (**Figure 1B**). As control, we analyzed seven infected C57BL/6J *Rag2^-/-^* mice (lacking functional B or T cells) from two independent experiments and did not find any such mutation in the GP33-41 epitope (**Figure 1A**). Together, these observations suggest that the viral mutations found in infected WT mice, although at relatively low frequencies, may have escaped the CTL response against the GP33-41 epitope.

The GP34A->T, which affects an anchor residue, and the GP36Y->C mutations were the most frequent mutations identified (four and three individual mice respectively) (**Figure 1C** and **Table 1**). We also detected the GP35V->A mutation, a known CTL escape mutation (**Figure 1C** and **Table 1**) (22, 41). Moreover, LCMV Clone 13 viruses containing either the GP34A->T or the GP36Y->C mutation were previously shown to persist in P14 mice, which encode a transgenic TCR specific for the GP33-41 epitope (9, 24), further suggesting that these mutations are likely to mediate escape from the GP33-41 specific CTL response.

**Table 1:** Identified mutations in the GP33-41 epitope and predictions of their impact on the binding strength between GP33-41 peptide and H2-D^b^.

Mutation	Identified in infected WT mice	Identified in WT mice with P14 TCR-transgenic splenocyte transfers	Identified in infected P14 TCR-transgenic mice	NetMHCpan prediction (%)
GP33-41 (WT)				0.391
**GP33K->E**	X			1.46
**GP33K->T**	X			0.395
**GP34A->T**	X	X	X	1.84
**GP34A->S**			X	0.868
**GP34A->V**			X	2.47
**GP35V->A**	X	X	X	0.585
**GP35V->I**	X			0.152
**GP36Y->C**	X		X	0.638
**GP36Y->F**			X	0.372
**GP37N->T**	X			6.00
**GP38F->I**	X			0.317
**GP39A->D**	X			0.182
**GP40T->S**	X			0.733

The predictions are the percentage rank scores calculated using the netMHCpan (v.4.0) prediction tool.

Next, we predicted the effect of the mutations on binding to H2-D^b^ by using the netMHCpan prediction tool (33). A score of <0.5% indicates strong predicted binding, a score of 0.5%-2% predicts weak binding whereas a score of >2% suggests that the peptide will not bind to the MHCI molecule (33). These analyses indicated a reduced H-2D^b^ binding strength of the three mutant peptides compared to WT peptide (**Table 1**).

### Infection of GP33-41-Specific TCR Transgenic Mice Resulted in the Rapid Emergence of Mutations in the Viral GP33-41 Epitope

**Figure 2:**
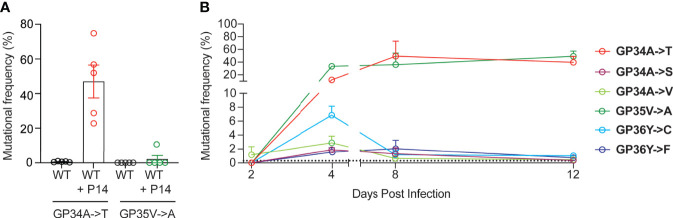
Increased GP33-41-specific CTL responses result in the rapid emergence of mutations in the viral GP33-41 epitope. **(A)** Frequency of mutations identified in the GP33-41 epitope at 40 days post infection in the WT mice that received splenocytes from P14 TCR-transgenic mice. The splenocytes were transferred one day before infection with LCMV CL13 WT. Five mice in each group were analyzed. **(B)** Mutations identified in P14 TCR-transgenic mice in the GP33-41 epitope using next-generation sequencing. The x-axis scale break indicates separate experiments (two total). Three mice were analyzed per timepoint. The dotted line indicates the limit of detection as defined in **Figure 1A**. In all graphs each error bar displays mean ± SEM.

In order to study the emergence of escape mutations in the GP33-41 epitope under increased selection pressure, we employed different approaches using P14 mice, which encode a transgenic TCR specific to the GP33-41 epitope (24). To increase CTL-mediated selection pressure on the GP33-41 epitope, we transferred 1 x 10^7^ splenocytes from P14 TCR-transgenic mice, which express the congenic CD45.1 marker, into WT mice. One day after splenocyte transfer the mice were infected with LCMV Clone 13 and harvested at 40 days post infection. As control, we observed the maintenance of P14 TCR-transgenic cells in the circulating CD8+ cell (CTL) population during the peak of the CTL response and during the chronic phase of infection, at eight and 39 days post infection respectively (**Supplementary Figure 2A**). Upon sequencing of viral RNA from spleens of these mice (**Supplementary Figure 3A**) we observed the GP34A->T mutation (**Figure 2A**), which we had previously identified in the WT mice (**Figure 1**). This mutation was observed at frequencies of at least 20% in all five of the splenocyte-transferred mice (**Figure 2A**). We also observed the GP35V->A mutation, however this was only detected in one out of five mice at a frequency of approximately 10% (**Figure 2A**). For both groups of mice, we obtained more than 8 x 10^3^ reads at the positions where the GP34A->T and the GP35V->A mutations occur (**Supplementary Figure 3B**). Further, we detected at least 2.5 x 10^3^ reads where either the GP34A->T or GP35V->A mutation was present in the mice that received splenocytes (**Supplementary Figure 3C**).

Next, we directly infected P14 TCR-transgenic mice to study the emergence kinetics of mutations in the GP33-41 epitope in response to near-monoclonal CTL selection pressure. Spleens of P14 TCR-transgenic mice were harvested between 1 and 39 days post infection. The CTL responses were analysed in infected P14 TCR-transgenic mice (**Supplementary Figures 2B, C**). Viremia and viral loads in spleen were also determined for these mice (**Supplementary Figures 4A, B**). Measurement of tetramer positive cells in the CD8 positive population of P14 TCR-transgenic mice confirmed that their CTLs recognized the GP33-41 epitope and did not recognize either of the immunodominant epitopes GP276-286 and NP396-404 (**Supplementary Figures 4C–E**).

Over the course of viral infection in P14 TCR transgenic mice, we detected six distinct mutations in the GP33-41 epitope (**Figure 2B**). GP35V->A and GP36Y->F were previously characterized as escape mutations (22, 41), while it was shown that viruses containing either the GP34A->T or GP36Y->C mutation cannot be cleared by P14 TCR-transgenic mice (9). The other mutations have not been reported in the literature so far. Intriguingly, the six observed mutations displayed different emergence kinetics (**Figure 2B**). GP34A->T and GP35V->A showed a large increase in frequency between two and four days post infection and maintained high frequencies from eight to 12 days post infection. In contrast the other four mutations increased in frequency from two to four days post infection but showed low frequencies at eight to 12 days post infection. These data suggest that GP34A->T and GP35V->A provided the greatest increase in viral fitness among the six identified nonsynonymous mutations in P14 TCR-transgenic mice.

We also assessed nonsynonymous mutations in the viral GP33-41 epitope of spleens from P14 TCR-transgenic mice that were infected 39 days previously. Interestingly, at this chronic time point we observed that the GP35V->A mutation was present at an average frequency of approximately 80% while the GP34A->T mutation showed significantly lower frequencies of below 20% (**Supplementary Figure 5A**). This was independently confirmed by Sanger sequencing using QSVanalyzer to perform semi-quantitative mutation frequency analysis (**Supplementary Figure 5B**) (34). This suggests that the GP35V->A mutation confers a relative fitness advantage over the GP34A->T mutation during chronic infection in the context of a near-monoclonal CTL selection pressure.

To investigate whether these mutations occurred within the same viral genomes, we analyzed the sequencing reads and observed that >99.8% of reads contained, mutually exclusively, either one of these mutations or none (**Supplementary Figure 6**). This indicated that the two mutations were not genetically linked but emerged in different viral genome molecules.

### The GP34A->T Mutation Changes the CTL Response Against the GP33-41 Epitope Without Impacting Viral Replication *In Vivo*

Next, we investigated the impact of the GP34A->T and GP35V->A mutations, which we had found in both WT mice and in our experimental systems with increased CTL selection pressure (**Figures 1** and **2**). To this end, we employed reverse genetically engineered LCMV Clone 13 strains containing either one of these mutations and, as control, the parental LCMV Clone 13 WT (26). Henceforth these viruses are referred to as CL13 GP34A->T, CL13 GP35V->A and CL13 WT respectively.

**Figure 3:**
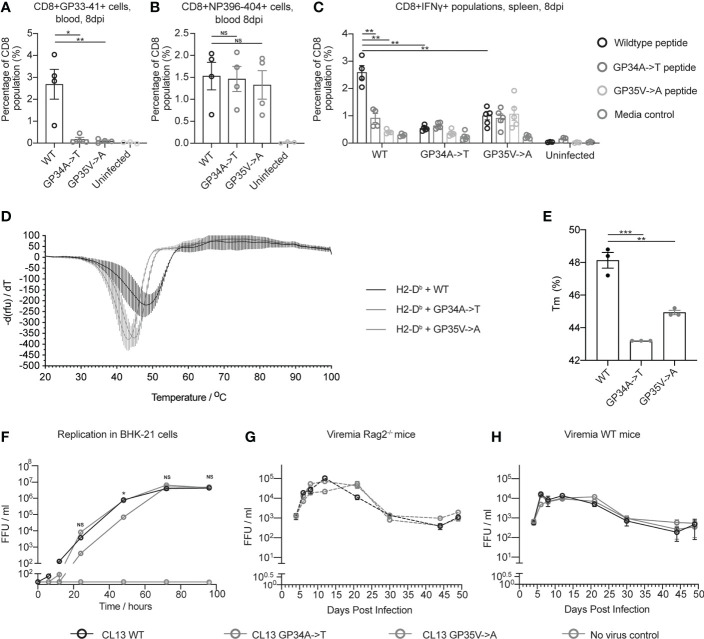
The GP34A->T mutation changes the CTL response against the GP33-41 epitope without impacting viral replication *in vivo*. The percentages of **(A)** GP33-41 and **(B)** NP396-404 tetramer positive cells in the circulating CD8+ CTL population were determined with flow cytometry at 8 days post infection. WT mice were infected with the indicated viruses. The uninfected control group are also shown. Between three and four mice were used per group. **(C)** The percentage of IFNγ positive cells in the splenic CD8+ population in WT mice infected with the indicated viruses and uninfected mice. Data were generated by intracellular cytokine staining at 8 days post infection and analysed with flow cytometry. Splenocytes were gated for the Lymphocyte/Single cell/Live/CD3+CD8+ population before being analyzed for IFNγ expression. Splenocytes from each mouse were stimulated with either GP33-41 WT peptide or peptide either containing the GP34A->T or GP35V->A mutation. Three to four mice were used per group. **(D)** Differential scanning fluorimetry measurements showing the melting temperature (Tm) of GP33-41 peptide-H2-D^b^ complexes. Negative first derivative of relative fluorescence units (rfu) plotted against increasing temperatures. The minimum point of the curves indicates the Tm of the corresponding peptide-H2-D^b^ complex. Three replicates were measured for each condition. **(E)** Bar graphs displaying Tm values of peptide-H2-D^b^ complexes, calculated from the curves in **(D)**. Each data point of the bar chart indicates the Tm for one technical replicate **(F)** BHK-21 cells were infected with a 0.01 multiplicity of infection (MOI) of the indicated viruses and media was harvested at the indicated timepoints. The scale break in the y-axis indicates the limit of detection. Data are representative of two independent experiments. Viremia from *Rag2^-/-^* **(G)** or WT **(H)** mice were infected with the indicated viruses. Four mice were analyzed per virus. The same mice were analyzed at each time point. Data are representative of two independent experiments. The scale break in the y-axis indicates the limit of detection. **(A–C, F–H)** each error bar displays mean ± SEM. In **(D–E)** the error bars indicate mean ± standard deviation. For **(A–C)** p-values were calculated using unpaired, two-tailed t-tests. For **(F)** p-values were calculated with one-way ANOVA using the Bonferroni correction. For **(A–C**, **F)** significant p-values are indicated as follows: *p ≤ 0.05, **p ≤ 0.01, ***p ≤ 0.001, NS, Not significant.

MHC class I tetramer staining revealed reduced numbers of GP33-41 specific CD8+ T cells in mice infected with CL13 GP34A->T or CL13 GP35V->A compared to the mice infected with CL13 WT (**Figure 3A**). Conversely, we found no difference in the percentage of CD8+ T cells specific for the NP396-404 epitope between mice infected with either of the three viruses (**Figure 3B**). These data indicate that the CTL response was normal in the mice infected with CL13 GP34A->T or CL13 GP35V->A, aside from the lack of a response to the GP33-41 epitope. These data indicate that the GP34A->T mutation escapes the anti-GP33-41 CTL response to the same degree as the GP35V->A mutation.

To investigate how the GP34A->T and GP35V->A mutations affect cytokine production of virus-specific CD8 T cells, we performed intracellular cytokine staining (ICS) upon peptide restimulation using splenocytes from mice infected with 2 x 10^6^ FFU of either CL13 WT, CL13 GP34A->T or CL13 GP35V->A. At eight days post infection, splenocytes were harvested and stimulated with WT GP33-41 peptide or GP33-41 peptides containing either the GP34A->T or the GP35V->A mutation (**Supplementary Figure 7**). We observed a smaller percentage of CD8+IFN*γ*+ cells when splenocytes from mice infected with CL13 WT were stimulated with either of the mutant peptides compared to the WT peptide (**Figure 3C**). We also observed that splenocytes of mice infected with either of the CL13 mutants showed a smaller percentage of CD8+IFNγ+ cells compared to splenocytes infected with CL13 WT upon stimulation with WT peptide (**Figure 3C**). Taken together these results support the conclusion that the GP34A->T and GP35V->A mutations escape the CTL response against GP33-41.

To experimentally assess the impact of the identified nonsynonymous mutations on peptide binding to MHCI, we performed differential scanning fluorimetry (DSF) (38, 40). Briefly, we performed UV-mediated peptide-exchange reactions with H2-D^b^-UVCP (ultraviolet light-cleavable peptide) complexes in the presence of WT or the mutant peptides. In agreement with our netMHCpan predictions (**Table 1**), we observed that both mutations (GP34A->T or GP35V->A) led to a decrease in the melting temperature of the GP33-41 peptide-H2-D^b^ complex and, hence, they led to the formation of less stable peptide-MHC complexes (**Figures 3D, E**).

We also applied DSF to measure the impact of the other mutations we observed in WT mice (**Figure 1A**) and P14 TCR-transgenic mice (**Figure 2B**), on the stability of the GP33-41 peptide-H2-D^b^ complex (**Supplementary Figure 8**). We found that eight out of the eleven tested peptides displayed similar melting temperatures to the GP33-41 WT peptide (**Supplementary Figures 8B, D–L**), indicating that these mutations do not affect the stability of the GP33-41 peptide-H2-D^b^ complex. In contrast, we observed that the GP33K->E mutation lowered the Tm of the GP33-41 peptide-H2-D^b^ complex compared to the complex containing the WT peptide (**Supplementary Figures 8B–C, L**). These results are in line with the netMHCpan predictions for this mutation as it leads to a drop in rank score (**Table 1**), suggesting that this mutation destabilizes the GP33-41 peptide-H2-D^b^ complex.

Further, we observed that the peptides with the GP36Y->C or the GP37N->T mutation (**Supplementary Figures 8M, N**), showed melting curves with no clear minima. This suggests that these peptides did not bind to, and consequently stabilize, the H2-D^b^ complex. These results are in line with the netMHCpan predictions as both mutations lead to a drop in the rank score, with GP33-41 peptide containing the GP37N->T mutation displaying the largest rank score of all the eleven analyzed peptides (**Table 1**), likely because it changes an anchor residue (**Figure 1C**). The GP36Y->C mutation, which arose in multiple WT mice (**Figure 1B**) and in P14 TCR-transgenic mice (**Figure 2B**) and has also been observed by Pircher et al. (9), providing accumulated evidence for conferring escape from GP33-41-specific CTL responses.

Upon elucidating the benefits endowed by the GP34A->T and GP35V->A mutations in regard to escaping the CTL response against GP33-41, we investigated whether either of these two mutations impose a replicative cost on the virus. To measure the replication of the three viruses *in vitro*, we infected BHK-21 cells and measured viral titers over a period of 96 hours. At 72 and 96 hours post infection all three viruses reached similar viral titers (**Figure 3F**). Yet, in two independent experiments we found that the virus containing the GP34A->T mutation grew slower between 24 and 48 hours post infection. To measure viral propagation in the absence of adaptive immune responses *in vivo*, we infected *Rag2^-/-^* mice with 2 x 10^6^ FFU of each of the viruses. We found no consistent difference in viremia in either *Rag2^-/-^* or WT mice infected with CL13 WT, CL13 GP34A->T or CL13 GP35V->A over the course of chronic infection (**Figures 3G, H**). Organ titers were also comparable for all three viruses in spleen, liver, kidney and brain of infected *Rag2^-/-^* and WT mice at 17 days post infection (**Supplementary Figure 9**). Taken together these data indicate that the mutations do not impose any replication cost on the virus nor confer a detectable increase in overall viral fitness by escaping one of many CTL epitope responses. This observation is in line with a previous study which showed that the presence of a single escape mutation in LCMV does not necessarily lead to impaired virus control in WT mice (9, 20).

## Discussion

CTLs are important to control chronic viral infections such as LCMV, HCV and HIV, which may evade this immune pressure by the selection of mutations in CTL epitopes (8, 9, 20, 22, 41–44). CTL escape mutations are linked to greater viral persistence and consequently with less favorable outcomes in both HIV and HCV (45, 46). In HIV, it has been shown that two general principles determine which escape mutations are selected for: the fitness cost that a mutation imparts upon the virus and how effective a mutation is at escaping the CTL response (15).

Here we focused on the immunodominant epitope GP33-41 during chronic LCMV infection and we employed a novel next-generation sequencing based approach to reveal the emergence of a variety of non-synonymous mutations at low frequencies.

The experimental increase of CTL-mediated selection pressure on a single viral epitope led to an accelerated emergence of epitope mutations and higher mutation frequencies. The fact that both the GP34A->T and GP35V->A mutations escape the CTL response to the GP33-41 epitope, while imposing little to no replication cost on the virus, suggests that these mutants would be selected for in a WT setting. This is in line with the fact that we identified both of these mutations in chronically infected WT mice. Despite the limited number of mice used in this study, we readily identified the aforementioned two mutations in both WT and P14 TCR-transgenic mice by next-generation sequencing at high coverage.

Using a viral infection model widely used to uncover fundamental aspects of CTL biology with relevance for human immunology (16, 18, 47, 48), we observed that CTL selection pressure selects a few distinct mutations from a pool of mutations in a CTL epitope. This may be applicable to chronic viral infection in humans, although mutations in CTL epitopes may show altered emergence kinetics that are determined by several parameters such as the individual’s MHCI haplotype and the immunodominance of the presented epitopes. Nonsynonymous mutations in CTL epitopes of acute infections such as SARS-CoV-2 may contribute to viral immune evasion (40).

In this study we focused on the selection of viral CTL escape mutations in the spleen. Yet, it is conceivable that other organ compartments may harbor organ-specific CTL escape mutations due to the local inflammatory milieu, immunoprivileged state, altered CTL responses and viral clearance kinetics during chronic infection (23, 49). While out of the scope of this study, such organ-specific differences present an interesting avenue for future investigations.

CTL escape mutations have also been reported in simian models of HIV after primates were vaccinated with vaccines containing a small number of CTL epitopes (50–52). A better understanding of how CTL escape mutations arise and precisely how these mutations interact with the immune system is an important consideration when designing and deploying CTL-based immunotherapies. Results from our study with the widely-used LCMV infection model combined with these observations in primates suggest that T cell-mediated vaccines against viruses should target a large number of CTL epitopes in order to avoid the development of CTL escape mutations.

We observed different emergence kinetics of the GP34A->T and GP35V->A mutations in the P14 TCR-transgenic mice compared to WT mice with P14 TCR-transgenic splenocyte transfer. In the P14 TCR-transgenic mice, the GP35V->A mutation accumulated to much higher frequencies than the GP34A->T mutation by 39 days post infection. This may be related to a small replicative disadvantage conferred by the GP34A->T mutation as suggested by our *in vitro* experiment, although further investigations are warranted to elucidate the involved mechanisms of viral intra-host competition.

However, in the context of P14 TCR transgenic splenocyte transfer the GP34A->T mutation was found at high frequency while the GP35V->A mutation was barely detectable. It is possible that the GP34A->T mutation escapes from the CTL response against the H2-K^b^ restricted GP34-43 epitope, which overlaps with the H2-D^b^ restricted GP33-41 epitope (53). It was shown that the GP35V->A mutation cannot escape the CTL response against the H2-K^b^ restricted GP34-43 epitope (22). While we observed that both the GP34A->T and GP35V->A mutations slightly decrease the stability of the GP34-43 peptide-H-2K^b^ complex (**Supplementary Figure 10**), it is possible that the GP34A->T mutation could mediate escape from the anti-GP34-43 response by reducing the recognition of the GP34-43 peptide-H2-K^b^ complex by CTLs. Such a mechanism of escape could explain why the GP34A->T mutation was selected for over GP35V->A in WT mice, as their CTLs recognize peptides presented by both H2-D^b^ and H2-K^b^ while CTLs of P14 TCR-transgenic mice recognize only the H2-D^b^ restricted epitope GP33-41.

It was shown that while GP33-41 peptide containing the GP35V->A mutation can be presented by H2-D^b^ and that CTLs can bind to this peptide-MHCI complex, this binding does not cause the proliferation of CTLs (54, 55). Lack of CTL proliferation due to the instability of the mutant peptide-H2-D^b^ complex is the likely mechanism of escape (54). We observed that both the GP35V->A and GP34A->T mutations led to a similar decrease in the thermostability of the GP33-41 peptide-H2-D^b^ complex, which suggests that the GP34A->T mutation may use a similar mechanism to escape from the CTL response against the GP33-41 epitope.

Using a novel next-generation sequencing based approach, we demonstrate that a variety of possible CTL escape mutations emerge in the LCMV genome during chronic infection and that specific mutations are selected for when immune selection pressure is increased. As chronic viral infections continue to be a substantial burden on human health, understanding how such mutations arise and impact immunobiology will be valuable for designing CTL-mediated vaccines and other immunotherapies against pathogen- or tumor-specific epitopes (15, 56, 57).

## Data Availability Statement

The next-generation sequencing data is available at the European Nucleotide Archive (ENA) with the accession number PRJEB41755.

## Ethics Statement

The animal study was reviewed and approved by Animal ethics committee of the Medical University of Vienna.

## Author Contributions

MS designed and executed most of the experiments. KK designed and executed some of the experiments and contributed to the design of experiments executed by MS. AP, LE, and MS analyzed the data for the next-generation sequencing experiment. VG, BA, AL, HB, LK, CV, and JH designed and/or conducted experiments. MS and AB wrote the manuscript with input from VG, BA, JH, LE, LK, and AL. AB supervised the study and experimental design. All authors contributed to the article and approved the submitted version.

## Funding

MS, KK, and AL were supported by DOC fellowships of the Austrian Academy of Sciences (No. 24813, No. 24158 and No. 24955 respectively). BA was supported by the Austrian Science Fund (FWF) DK W1212. VG and JH received support from the Vienna Science and Technology Fund (WWTF, LS14-031).

## Conflict of Interest

The authors declare that the research was conducted in the absence of any commercial or financial relationships that could be construed as a potential conflict of interest.
