# Supplementary material for: Characterization of CD8 T Cell-Mediated Mutations in the Immunodominant Epitope GP33-41 of Lymphocytic Choriomeningitis Virus
Source: Front Immunol. 2021 Jun 14;12:638485. doi: 10.3389/fimmu.2021.638485 (PMC8236698; doi:10.3389/fimmu.2021.638485)
Supplement: Supplementary file 1 [file DataSheet_1.pdf]

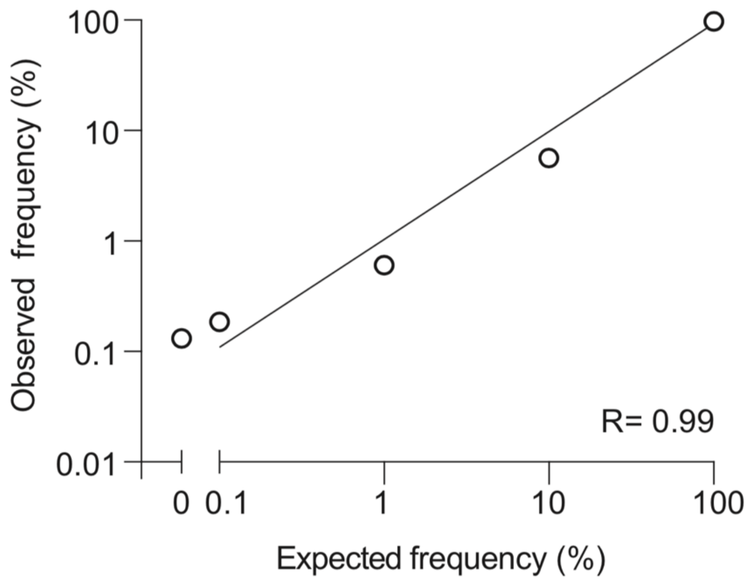

Supplementary Figure 1. Validation of mutation calling by amplicon-based next-generation sequencing. A plasmid containing the LCMV S segment with the GP34A->T mutation was serially diluted in a solution of plasmid containing the LCMV WT S segment to obtain the indicated allele frequencies of the mutant plasmid. Samples were sequenced and the frequencies of the GP34A->T mutation were calculated. The correlation coefficient, R, is displayed on the graph. Each data point represents one sample.

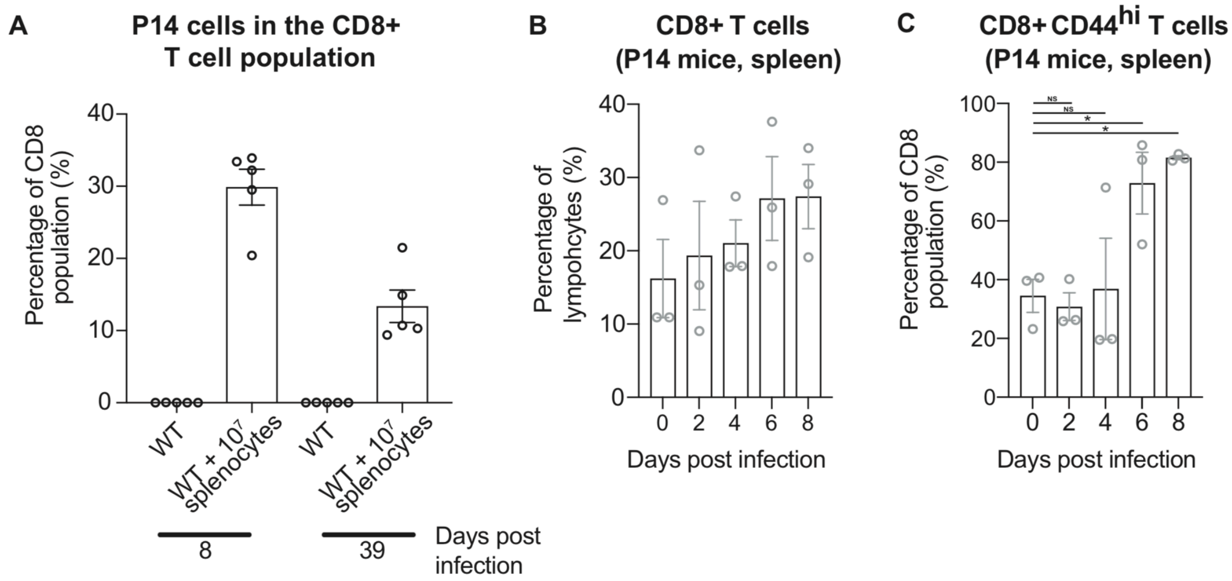

Supplementary Figure 2. The number of CTL (CD8+) cells specific for GP33-41 was experimentally increased to modulate the selection pressure on the GP33-41 epitope. (**A**) The percentage of P14 TCR-transgenic cells (CD45.1 expressing) in the CD8+ population in the blood of WT mice with 1 x 10^7 splenocytes transferred from P14 TCR-transgenic mice, or with no transfer, at 8 and 39 days post infection. Five mice were analyzed in each group. The same mice were analyzed at each timepoint. The percentage of (**B**) CD8+ and (**C**) activated CTL (CD8+CD44^{hi}) cells in the (**B**) splenic lymphocyte or (**C**) CD8+ population in P14 TCR-transgenic mice infected with LCMV CL13 WT. The same three mice were analyzed at each timepoint in (**B**) and (**C**) (15 mice total). In all graphs the percentages were calculated from flow cytometry data. Lymphocytes were gated for the Lymphocyte/Single cell/Live population in (**A**) and (**C**) and for the Single cell/Live/CD8+ population in (**B**) before being further analyzed. P-values were calculated using unpaired, two-tailed t-tests. Significant p-values are indicated as follows: *= $p \leq 0.05$, NS= not significant. For all graphs each error bar displays mean ± SEM.

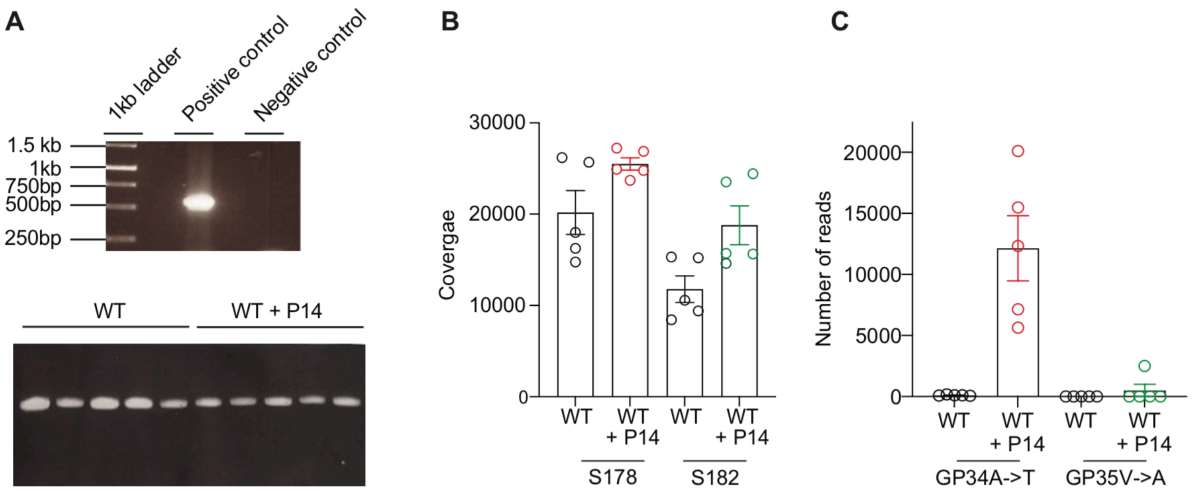

Supplementary Figure 3. The GP34A->T and GP35V->A mutations in the splenocyte transfer mice were supported by a large number of next-generation sequencing reads. All data are from the same experiment as shown in Figure 2A. **(A)** Electrophoresis gel pictures of 10ul of the PCR reactions that were sequenced with next-generation sequencing. One band relates to one mouse**.** The control and test samples were run on different gels (top and bottom pictures respectively) and photographed with different exposures and magnifications. Both the test samples and control samples are from one PCR reaction. **(B)** The number of next-generation sequencing reads at positions 178 and 182 in the S segment. The GP34A->T mutation arises at the S178 position and the GP35V->A mutation arises at the S182 position. All sequencing was done from spleen samples. All mice were infected with LCMV CL13 WT and sacrificed at 40 days post infection. There were five mice analyzed per group. **(C)** The number of next-generation sequencing reads containing either the GP34A->T or GP35V->A mutation.

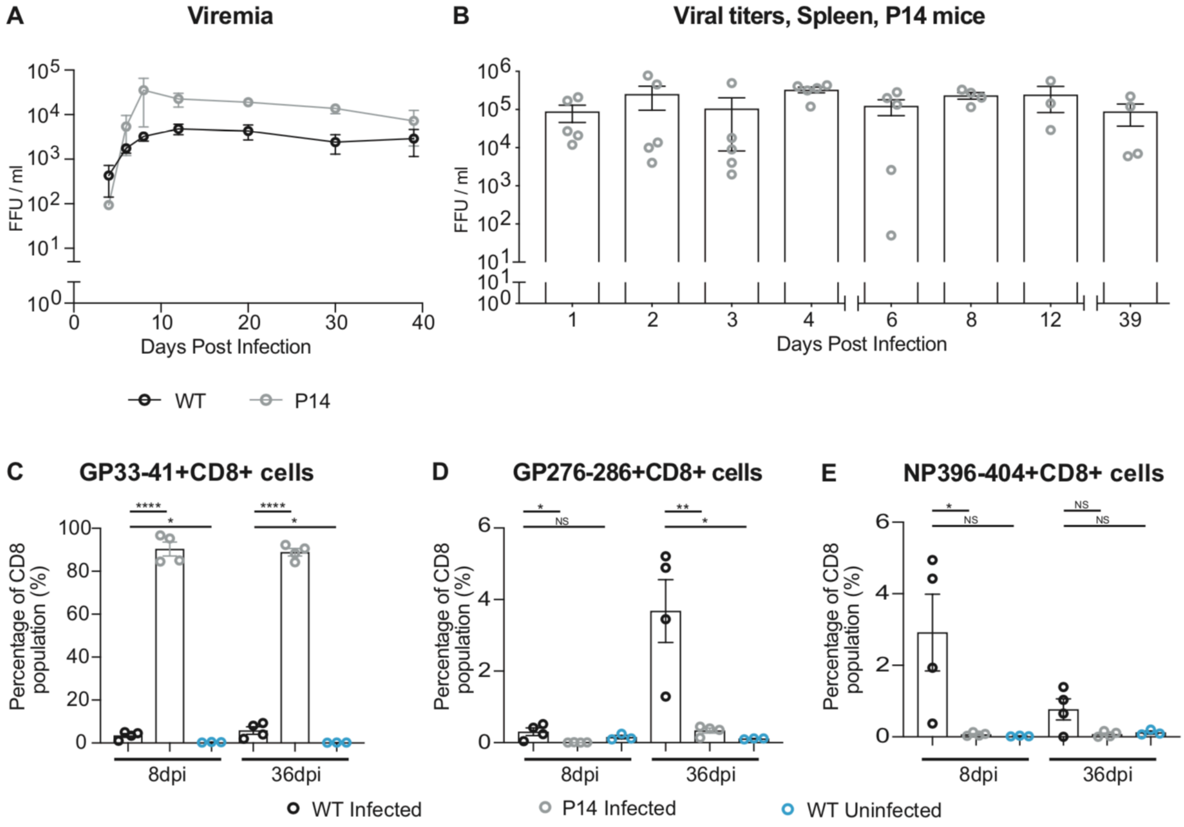

Supplementary Figure 4. P14 TCR-transgenic mice show detectable viral titers in the spleen and in blood during chronic LCMV infection. Viral titers in (**A**) blood and (**B**) spleen of WT and P14 TCR-transgenic mice infected with LCMV CL13 WT were quantified using focus forming assay over the course of chronic infection. Three to five mice were used per timepoint. X-axis scale breaks in (B) indicate separate experiments. The percentage of (**C**) GP33-41 tetramer positive, (**D**) GP276-286 tetramer positive and (**E**) NP396-404 tetramer positive cells in the circulating CD8+ population at 8 and 39 days post infection (dpi). WT and P14 TCR-transgenic mice were infected with LCMV CL13 WT. (**C-E**) Cells were gated for the Lymphocyte/Single cell/Live/CD8+ population before being analyzed for tetramer binding. Three to four mice were used per group. The same mice were measured at each timepoint. Significant p-values are indicated as follows: *= p≤0.05, **= p≤0.01, ****= p≤0.0001 NS= not significant. For all graphs each error bar displays mean ± SEM.

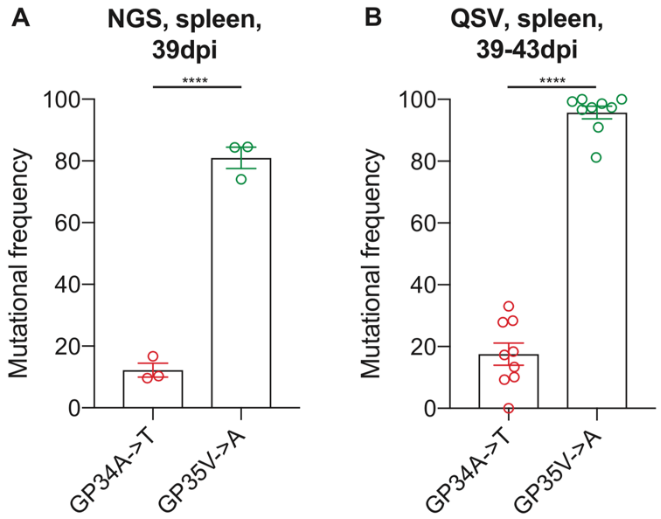

Supplementary Figure 5. Comparative analysis of the frequencies of the GP34A->T and GP35V->A mutations calculated using data from next-generation and Sanger sequencing at 39 dpi (days post infection) and 39-43dpi respectively. (**A**) The frequency of the GP34A->T and GP35V->A mutations calculated from NGS reads. The spleens of three P14 TCR-transgenic mice were analyzed and the frequencies of both mutations were calculated for each mouse. (**B**) The frequencies of the GP34A->T and GP35V->A mutations were quantified from Sanger sequencing tracks using QSVanalyzer (35). The spleens of nine P14 TCR-transgenic mice from three separate experiments were analyzed in total, including the three mice shown in (**A**). Error bars display mean ± SEM. P-values were calculated using unpaired, two-tailed t-tests. Significant p-values are indicated as follows: ****= p≤0.0001.

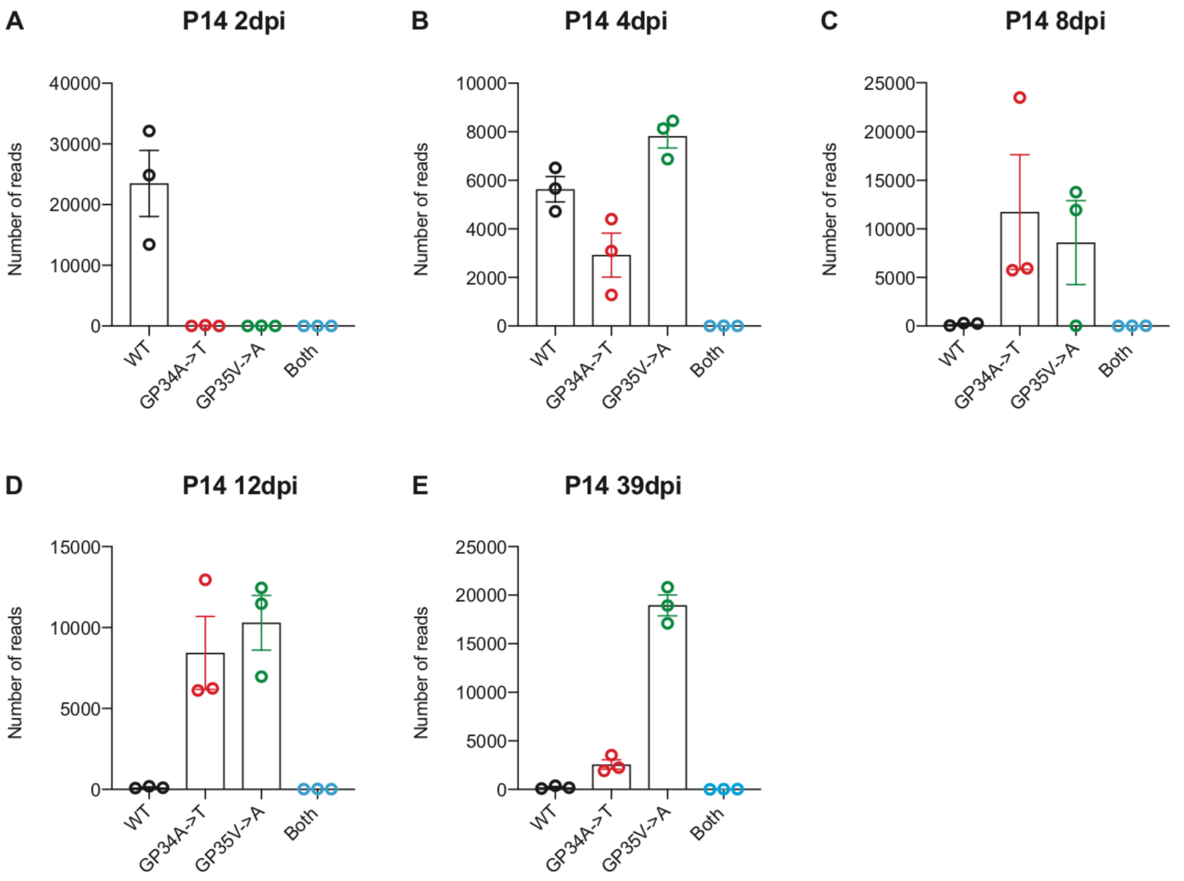

Supplementary Figure 6. GP34A->T and GP35V->A mutations occur mutually exclusively on the same genome. The number of reads where the GP33-41 epitope contains neither the GP34A->T nor the GP35V->A mutation, only the GP34A->T mutation, only the GP35V->A mutation or both the GP34A->T and the GP35V->A mutations from the spleens of P14 TCR-transgenic mice at (**A**) 2 days, (**B**) 4 days, (**C**) 8 days, (**D**) 12 days and (**E**) 39 days post infection. The reads analyzed were from the next-generation sequencing shown in Figure 2B. Samples from three mice were sequenced and each data point represents reads from one mouse. Error bars display mean ± SEM.

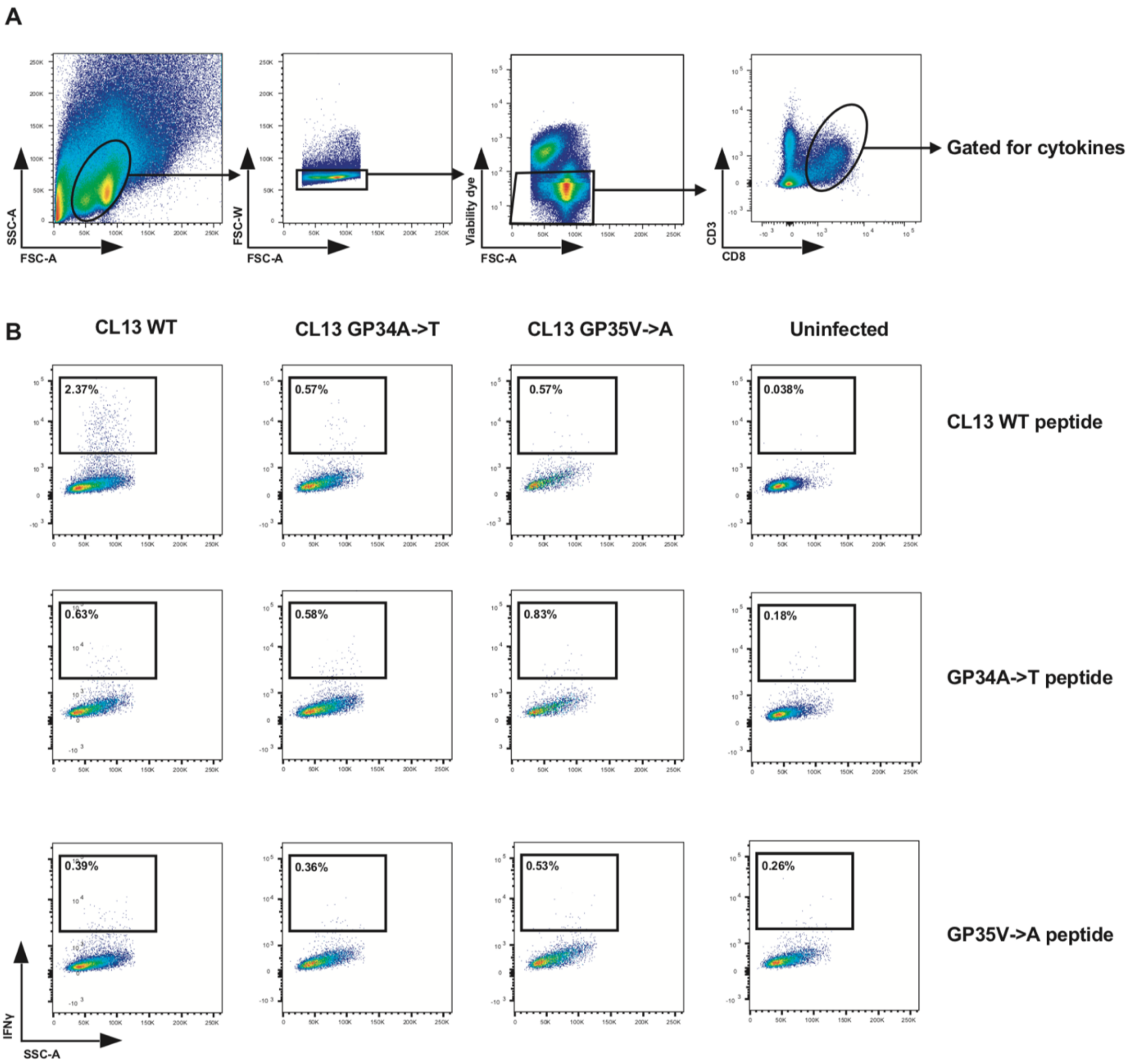

Supplementary Figure 7. Gating strategy for the intracellular cytokine staining experiment. (**A**) Representative flow cytometry plots indicating the gating strategy. (**B**) Single, representative flow cytometry plots for the intracellular cytokine staining experiment graphed in Figure 3C. Each plot represents one of the possible combinations of viral infection (LCMV CL13 WT, LCMV CL13 GP34A->T, LCMV CL13 GP35V->A and no infection) and stimulating peptide (GP33-41 WT peptide, GP33-41 peptide containing the GP34A->T mutation and GP33-41 peptide containing the GP35V->A mutation). Splenocytes were gated for the Lymphocyte/Single cell/Live/CD3+CD8+ population before being analyzed for IFNγ expression. The percentages of IFNγ positive cells are shown in the top left hand corner of the plots.

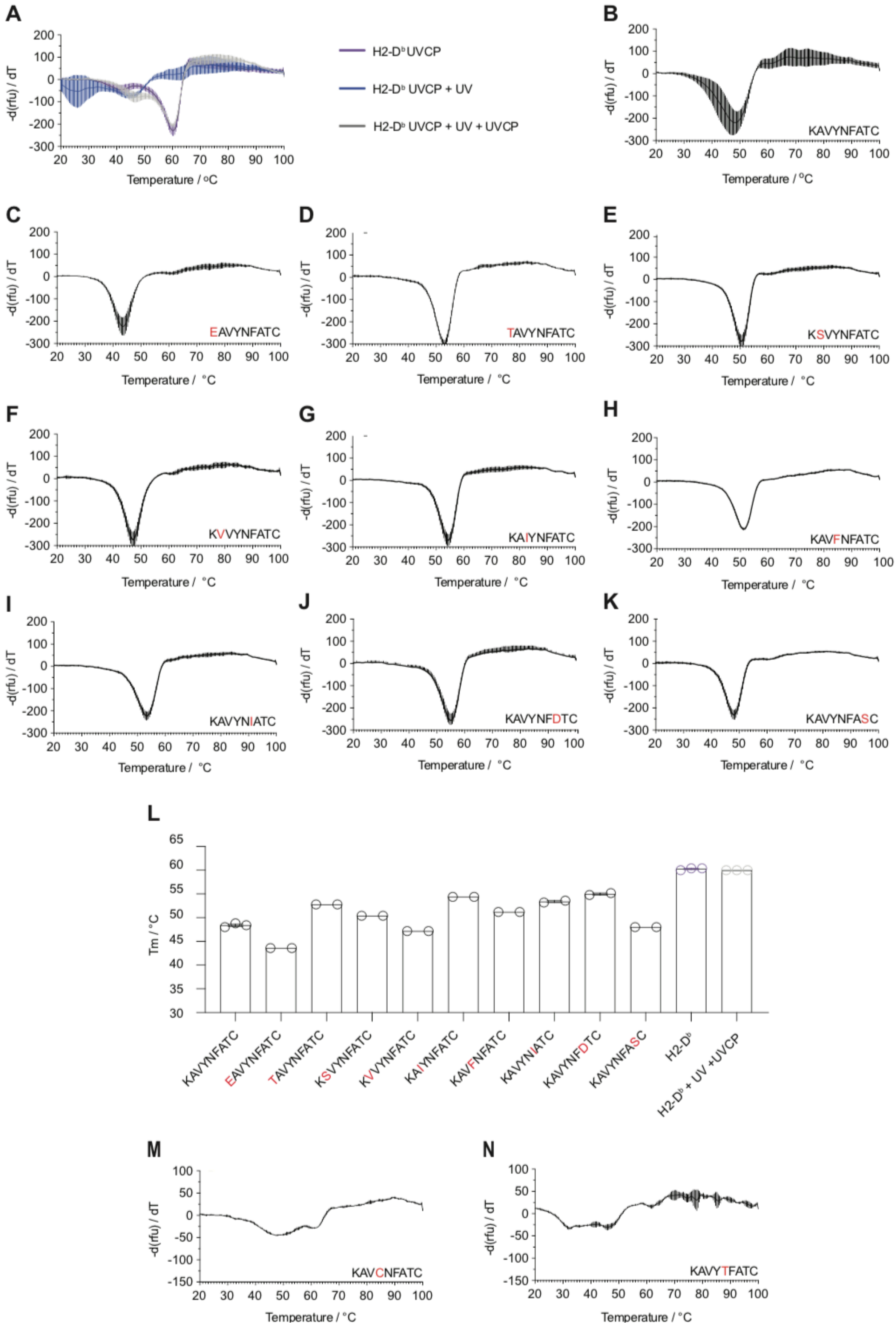

A
B
C
D
E
F
G
H
I
J
K
L
M
N
H2-Dᵇ UVCP
H2-Dᵇ UVCP + UV
H2-Dᵇ UVCP + UV + UVCP
-d(rfu) / dT
Temperature / °C
Tm / °C
KAVYNFATC
EAVYNFATC
TAVYNFATC
KSVYNFATC
KVVYNFATC
KAIYNFATC
KAVFNFATC
KAVYNIATC
KAVYNFDTC
KAVYNFASC
H2-Dᵇ
H2-Dᵇ + UV +UVCP
KAVCNFATC
KAVYTFATC

Supplementary Figure 8. The GP33K->E, GP36Y->C and GP37N->T mutations destabilize the GP33-41 peptide-H2-D^b complex. (A-K) Graphs display melting curves calculated with differential scanning fluorimetry (DSF). The minima of any given curve along the x-axis indicates the melting temperature (Tm) of the peptide-H2-D^b complex. All data is representative of at least two DSF measurements and error bars indicate mean ±standard deviation. **(A)** Melting curves of control peptides. The purple curve represents H2-D^b UVCP (UV cleavable peptide) that is not exposed to UV light. The blue curve represents H2-D^b UVCP which has been exposed to UV light. The grey curve represents H2-D^b UVCP to which UVCP was added after exposure to UV light. (**B-K**) Melting curves represent H2-D^b UVCP which has been exposed to UV light in the presence of GP33-41 WT peptide (B) or mutant peptides (C-K). (**L**) Bar graphs displaying Tm values of peptide-H2-D^b complexes calculated from the curves in (**A-K**). Each data point of the bar indicates the Tm for one technical replicate and the error bars indicate mean ±standard deviation. (**M-N**) Melting curves represent H2-D^b UVCP which has been exposed to UV light in the presence of the respective peptide.

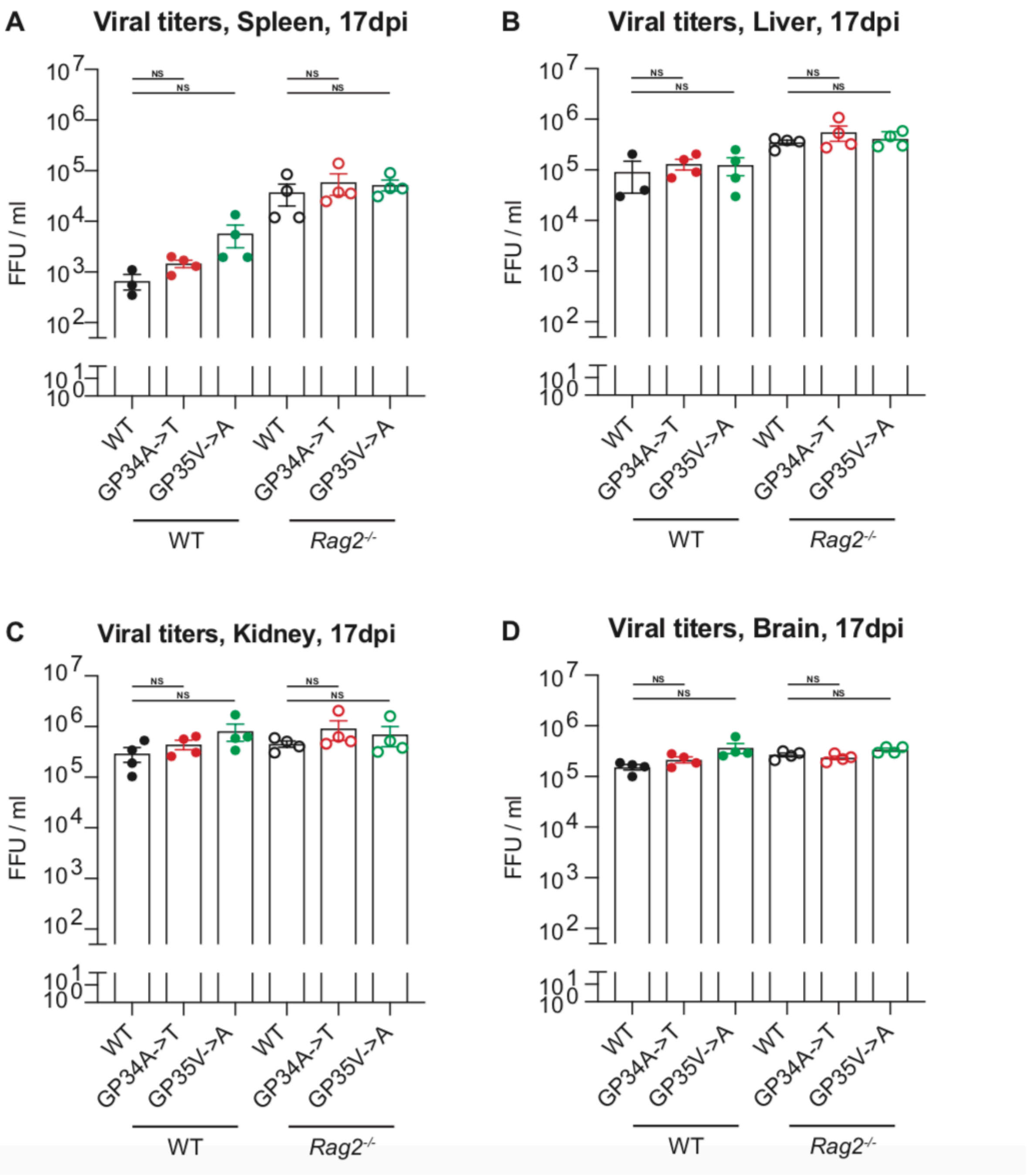

Supplementary Figure 9. Viral titers of LCMV CL13 WT, LCMV CL13 GP34A->T and LCMV CL13 GP35V->A at 17 days post infection in solid organs. Organ titers from (**A**) spleen, (**B**) liver, (**C**) kidney, and (**D**) brain. Mice of the indicated genotype were infected with the respective viruses. All titers were quantified with focus forming assay and three to four mice were used per group. In all graphs the scale break in the y-axis indicates the limit of detection. Error bars display mean ±SEM and p-values were calculated using unpaired, two-tailed t-tests. Significant p-values are indicated as follows: NS= not significant.

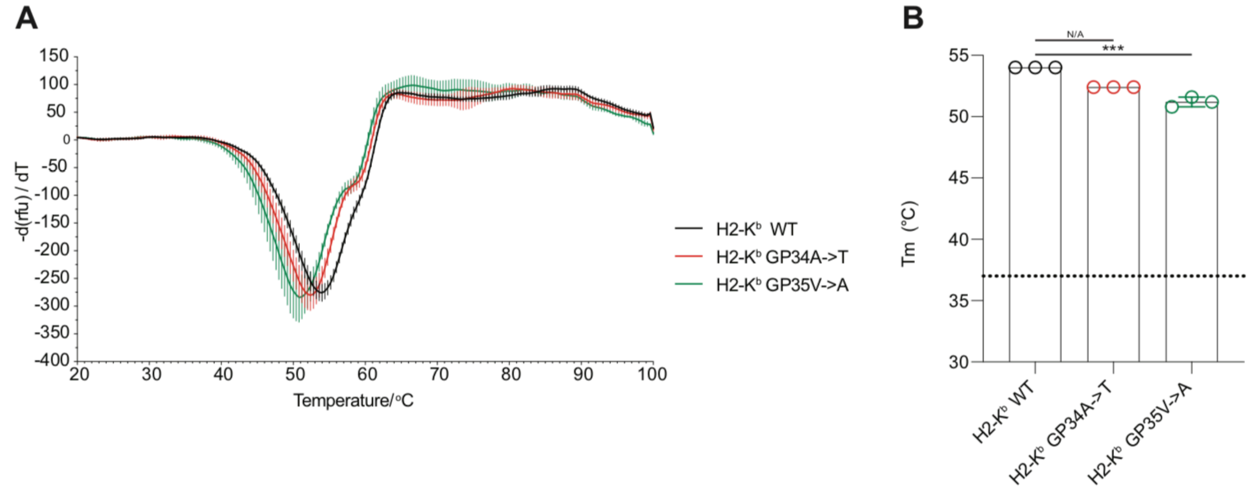

Supplementary Figure 10. The GP34A->T and GP35V->A mutations do not affect the binding of the GP34-43 epitope to H2-K^b. (**A**) Melting curves of H2-K^b complexes with wildtype and mutant GP34-43 peptides. The lowest point of any given curve along the x-axis indicates the Tm of the peptide-H2-K^b complex. Three replicates were measured for each condition and error bars indicate mean ± standard deviation. (**B**) Melting temperatures (Tm) of the peptide-H2-K^b complexes calculated from the graph in (**A**). Each data point represents the Tm of one technical replicate and error bars indicate mean ± standard deviation. Significant p-values are indicated as follows: ***= p≤0.001 and N/A= Not available (can't be calculated).
